# Asymptomatic bacteriuria and urinary tract infection in geriatric inpatients after indwelling urinary catheter removal: a descriptive two-centre study

**DOI:** 10.1016/j.infpip.2024.100411

**Published:** 2024-10-24

**Authors:** Aysel Kulbay, Eva Joelsson-Alm, Karin Amilon, Ann Tammelin

**Affiliations:** a Department of Medicine, Solna (MedS), Unit of Infectious Diseases, Karolinska Institutet, Stockholm, Sweden; b Department of Clinical Science and Education, Södersjukhuset, Karolinska Institutet, Stockholm, Sweden; c Department of Anaesthesiology and Intensive Care, Södersjukhuset, Stockholm, Sweden; d Department of Laboratory Medicine, Division of Clinical Microbiology, Karolinska Institutet, Stockholm, Sweden; e Department of Clinical Microbiology, Karolinska University Hospital, Stockholm, Sweden

**Keywords:** Asymptomatic bacteriuria, Indwelling urethral catheter, Geriatric post-void residual urine volume

## Abstract

**Background:**

Patients with indwelling urinary catheters (IUC) are common in geriatric care. Catheterization increases the risk of asymptomatic bacteriuria (ASB) and urinary tract infection (UTI). The prevalence of ASB after IUC-removal is only sparsely studied. This study aimed to compare the occurrence of ASB and UTI in geriatric patients with and without a history of catheterization and to explore factors associated with ASB.

**Methods:**

Patients were included at two geriatric rehabilitation wards in Stockholm, Sweden. Data were collected about history of catheterization, antibiotic treatment, and diabetes mellitus. Urine samples were analysed. Occurrence of UTI during inpatient care was identified by patient records.

**Results:**

In total 196 asymptomatic patients were included in the analysis. Asymptomatic bacteriuria was significantly more common in patients with a history of catheterization (38/104, 36.5%) compared to those without IUC during the past four weeks (19/92, 20.6%, *P*=0.018). Enterococci were more commonly found in patients with a history of catheterization. Of 124 patients possible to follow up, five UTI-cases were found during hospital stay. All cases had had ASB and 4/5 had had an IUC on admission.

Catheterization was significantly associated with ASB after adjustment for confounders (OR 2.79, CI 1.31–5.91, *P=*0.008).

**Conclusions:**

Catheterization is associated with ASB, this persists after IUC-removal. The results indicate that colonisation by *Enterococcus* species linked to catheterization may persist for at least four weeks after IUC-removal.

**Trial registration:**

The study is registered at clinicaltrials.gov with the identification number NCT05039203 (09/09/2021).

## Introduction

An indwelling urinary catheter (IUC) impairs the bladder's normal host defence by causing mucosal inflammation which results in tissue damage, allowing colonisation by bacteria [[1], [2], [3]]. Prolonged catheterization increases the risk of both asymptomatic bacteriuria (ASB) and urinary tract infection (UTI) [[4], [5], [6], [7]]. In a randomised controlled trial on inpatients, Warren *et al.* [8] found that the incidence of IUC-associated ASB was increased with 5% for each day of catheterization. Notably, ASB has been found in all patients catheterized for ≥30 days [[9], [10], [11], [12]].

Without catheterization, prevalence of ASB is higher in the elderly (women aged 80 years 15–20%, men aged 80 years 5–10%) than in young healthy adults (women <5%, men <0.1%) [[13], [14], [15], [16]]. Higher prevalence of ASB is also found in diabetic women (9–27%) compared to diabetic men 0.7–1% [12]. The highest prevalence of ASB is found in elderly residents of nursing homes (women 25–50%, men 15–50%) [[17], [18], [19]].

Geriatric patients are at high risk of healthcare associated UTI due to catheterization as they often have other risk factors such as chronic diseases, elevated (>200mL) post-void residual urine volume (PVR) and altered immune function [20,21].

Asymptomatic bacteriuria and UTI in catheterized patients are well studied but less is known about what happens after IUC-removal [[22], [23], [24], [25]].

The aim of this study was to compare the occurrence of ASB in voided urine samples and thereafter the first occurrence of an UTI-event in geriatric patients with and without a history of catheterization and to explore factors associated with ASB.

## Methods

### Study population

This study was conducted at two departments of geriatric rehabilitation in Stockholm, Sweden. The participating two wards had about 40 beds each and were profiled on rehabilitation after orthopaedic surgery and rehabilitation after stroke in patients aged ≥65 years. The study period was from May 2022 to February 2024.

All patients admitted to the wards were eligible for study participation ≤48 hours after admission. The inclusion criteria were ability to understand oral and written instructions in Swedish and ability to produce a voided urine sample. Exclusion criteria were severe cognitive disability, physical disability to produce voided urine samples, end-of-life care, and ongoing antibiotic treatment for UTI. Patients fulfilling the inclusion criteria received oral and written information about the study. Written consent was provided from all participants before inclusion. Study participants with symptoms or signs of UTI were examined by a physician. Patients with confirmed UTI-diagnosis according to the EAU-guidelines for urological infections [26] were excluded from the study. UTI-cases were excluded at baseline to be able to evaluate the participants for ASB. Patients receiving an IUC during the study period were also excluded.

### Definitions

Criterium for diabetes mellitus was any kind of treatment, i.e., diet only, oral medication and/or insulin treatment.

Asymptomatic bacteriuria was defined as a uropathogen in a quantity of ≥10^5^ CFU/mL [27] detected in a voided urine sample from a person without symptoms or signs of UTI.

*Escherichia coli* and *Staphylococcus saprophyticus* were regarded as primary uropathogens. Secondary uropathogens included *Enterococcus* species, *Enterobacter* species, *Klebsiella* species, *Proteus mirabilis*, *Morganella morganii, Pseudomonas aeruginosa, Staphylococcus aureus* and *Citrobacter* species. Tertiary uropathogens included coagulase-negative staphylococci*, Streptococcus agalactiae* (Group B streptococci) and *Candida* species [28,29].

Bacteriuria according to recommendations for diagnosis of UTI included primary uropathogen ≥10^3^ CFU/mL, secondary uropathogen as single species ≥10^3^ CFU/mL, secondary uropathogen ≥10^5^ CFU/mL when not as single species, and tertiary uropathogen as single species ≥10^5^ CFU/mL [28,29].

History of catheterization included patients with an IUC on admission or with an IUC present at any time during the past four weeks prior to admission irrespective of the date of the IUC-insertion.

The criterium for the first UTI-event occurring during the inpatient period after the initial urine sampling for detection of ASB was antibiotic treatment for UTI.

A PVR of >200 mL was regarded as elevated.

### Data and sample collection

A structured questionnaire with 10 questions, constructed by the researchers, was used. Five questions concerned catheterization at any time during the past four weeks prior to admission or at admission, the reason for catheterization when applicable and date of IUC-removal. Three questions concerned the reason for antibiotic treatment and duration of any ongoing or past antimicrobial treatment within the last two weeks and the presence of diabetes mellitus. Two questions concerned new or worsened symptoms from the urinary tract. Face validity was tested prior to the study start using think aloud technique [30]. Eight persons (3 women, 5 men) ≥65 years and 10 healthcare personnel (8 nurses, 2 physicians) answered the questionnaire individually. This led to a slight change of the wordings to distinguish new or worsened symptoms from the urinary tract from chronic genitourinary symptoms. Patients' records were used to complete information in the questionnaires.

One voided urine sample was collected from each non-catheterized patient on admission, directly after study inclusion. For catheterized patients on admission, IUC-removal could be performed close to admission or later on during the inpatient care when the patients were sufficiently mobilised, depending on the physician's decision. From these patients, a voided urine sample was collected as soon as possible after IUC-removal, preferably on the same day. After voiding, all study participants had their PVR measured with a portable ultrasonic bladder scanner. Data was collected from medical records about the first UTI-event occurring during the inpatient period after the initial sampling for detection of ASB.

### Microbiological analysis

The urine samples were cultured at the microbiological laboratory in Karolinska University Laboratory, Karolinska University Hospital, Region Stockholm, Sweden. Bacterial isolation and identification were performed by routine methods at the laboratory. Briefly, 10 μL of urine was inoculated onto blood-, chromogenic UTI- and Mueller Hinton Fastidious (MHF) agar. Antibiotic discs for the most common antibiotics used for treatment of UTI were placed on MHF agar plates. Plates were incubated for a minimum of 18 hours, after which bacterial growth was assessed and quantified. Species identification was performed by a combination of morphology, species-specific antibiotic susceptibility patterns, and MALDI-TOF mass-spectrometry analysis.

### Statistical analysis

Continuous variables were expressed as medians (IQR) and categorical variables as frequencies (percentage). Depending on the data, either Chi-square test, Fischer's exact test or Mann-Whitney U test was used to compare differences between the groups.

Binary logistic regression was used to test associations with ASB. We categorised four explanatory variables into two groups each: age in years (under 80 yrs./80 yrs. or older), sex (woman/man), diabetes mellitus (yes/no), IUC on admission or at any time during the past 4 weeks before admission (yes/no), PVR (200 mL or less - more than 200 mL). One explanatory variable was categorised into three groups: antibiotic treatment (1. no antibiotic treatment on admission or two weeks before urine sample, 2. antibiotic treatment on admission, 3. no antibiotic treatment on admission, but within 2 weeks before urine sample).

Univariable analysis was performed to study crude associations of each explanatory variable with ASB. Variables with a *P*-value ≤0.2 in the univariable analyses were included in the multivariable analyses. Multivariable logistic models were used to study the adjusted associations presented as odds ratios (OR) with 95% confidence intervals (CI). To test the final adjusted model, Hosmer-Lemeshow goodness of fit test was used, where *P*-value above 0.05 indicated an acceptable fit.

The IBM Statistical Package for the Social Sciences (SPSS) version 28.0 (IBM Corp., Armonk, NY, USA) was used for statistical analyses and a two-sided *P*-value of <0.05 was considered statistically significant in the final analyses.

### Ethics approval and consent to participate

Ethical approval was granted by the Swedish Ethical Review Authority (registration number 2021–02410, 2022-02915-02 and 2022-06567-02). All study participants provided a written informed consent and were able to end the study participation at any time. The recruitment of participants followed the Declaration of Helsinki [31].

## Results

### Participants

**Figure 1:**
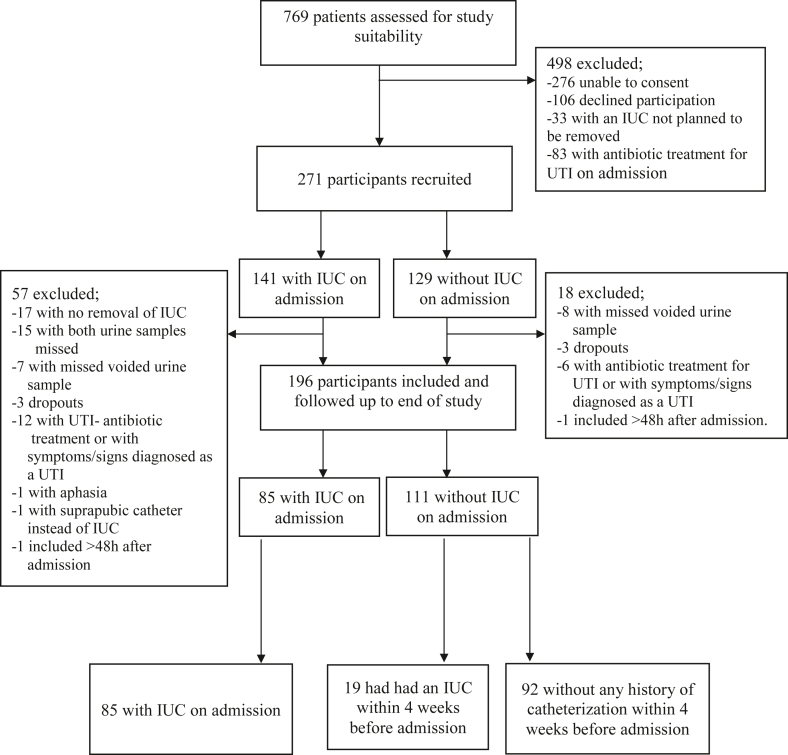
Flowchart of study inclusion process.

A total of 271 patients were recruited for the study of whom 75 patients were excluded (57 from the group with IUC on admission and 18 from the group without IUC on admission) (Figure 1). The excluded patients did not differ significantly regarding age group (*P*=0.164), or sex (*P*=0.775) compared to the included 196 patients.

Among the included patients, 85 had an IUC in place on admission and 111 did not. Among those with an IUC, the catheterization had mostly been initiated at an acute care hospital within one month prior to admission and only three patients had started the catheterization more than one month earlier. In the group without IUC, 19 patients had had an IUC at some time during four weeks before admission. Hence, 104 patients had a history of catheterization on or within four weeks of admission (Table I).

**Table I:** Characteristics of study participants

Variable, n (%)	IUC in place on admission, n=85	No IUC in place on admission and no history of catheterization during past 4 weeks before admission, n=92	No IUC in place on admission but with history of catheterization during past 4 weeks before admission, n=19
Sex			
Women	50 (59%)	68 (74%)	11 (58%)
Men	35 (41%)	24 (26%)	8 (42%)
Age in years, median (IQR)	83 (77–80)	81 (76–87)	80 (75–89)
Indication for catheterization			
Urinary retention	32 (38%)	-	2 (10%)
Perioperative routine	31 (36%)		14 (74%)
Other reasons	22 (26%)		3 (16%)
Start of catheterization			
Less than 2 weeks ago	75 (88%)	-	16 (84%)
2–4 weeks ago	7 (8%)		3 (16%)
More than 1 month ago	3 (4%)		0 (0%)
Time from IUC-removal to voided urine sampling			
Same day as IUC-removal	67 (79%)	-	1 (5%)
1 day after IUC-removal	8 (9%)		4 (21%)
2 days after IUC-removal	2 (2%)		4 (21%)
3 days after IUC-removal	2 (2%)		3 (16%)
4 days or more after IUC-removal	6 (7%)		7 (37%)
Diabetes mellitus			
Yes	32 (38%)	22 (24%)	5 (26%)
No	53 (62%)	70 (76%)	14 (74%)
Antibiotic treatment			
No antibiotic treatment on admission or within two weeks prior to urine sample	36 (42%)	74 (80%)	13 (68%)
Antibiotic treatment on admission	29 (34%)	15 (16%)	3 (16%)
No antibiotic treatment on admission, but antibiotic history within 2 weeks prior to urine sample	20 (24%)	3 (3%)	3 (16%)
PVR in mL, median (IQR)	111 (48–226)	78 (26–180)	138 (33–190)
PVR			
≤200 mL	59 (69%)	71 (77%)	16 (75%)
>200 mL	26 (31%)	19 (21%)	3 (16%)
Missing		2 (2%)	

IQR Interquartile range.

IUC Indwelling urethral catheter.

UTI Urinary tract infection.

PVR Post-void residual urine volume.

The median age of the 196 participants was 81 years. Most of them were women (129/196, 65.8%). Presence of an IUC on admission or at any time during the past four weeks before admission was significantly more common in men (43/67) than in women (61/129) (*P=*0.034).

Diabetes mellitus did not differ significantly between the patients with or without IUC on admission nor in patients with a history of IUC before admission (*P=*0.128).

Of the 196 patients, 123 (62.8%) had no ongoing or past antibiotic treatment within two weeks prior to urine sample. Ongoing antibiotic treatment for an infection or as perioperative prophylaxis was significantly more common in patients with an IUC on admission or during the past four weeks before admission (55/104) compared to patients without history of catheterization (18/92) (*P*<0.001).

The PVR was measured in 194 of 196 patients. The median PVR in mL was equally distributed among the patients with or without IUC on admission or with a history of IUC at any time during the past four weeks (*P=*0.094). There was no statistically significant difference in PVR (≤200 mL/>200 mL) in patients with or without IUC on admission nor in patients with a history of IUC prior to admission (*P=*0.221) (Table I).

### Asymptomatic bacteriuria and uropathogens

Asymptomatic bacteriuria was found in 57 patients. It was significantly more common in the 104 patients with a history of catheterization than in the 92 patients without IUC on admission nor any prior IUC during the past four weeks (38/104 vs 19/92, *P=*0.018). Urine samples from the patients with an IUC on admission were mostly obtained on the same day as IUC-removal whereas samples from the patients with a history of catheterization during the past four weeks mostly were obtained four or more days after IUC-removal (Table I). There was however no significant difference in ASB prevalence irrespective if the urine was sampled within the same day of IUC-removal, one, two, three or four or more days after removal (*P=*0.399).

The prevalence of ASB did not differ significantly between men (14/67) and women (43/129) (*P=*0.097) nor between diabetic (22/57) and non-diabetic patients (35/57) (*P=* 0.123). Of the 59 patients in the study with diabetes, significantly more diabetic women (16/31) had ASB compared to diabetic men (6/28) (*P=* 0.030).

Bacteriuria in the quantity of 10^3^–10^5^ CFU/mL was equally distributed among the patients with or without IUC on admission or with a history of catheterization during the past four weeks (Table II).

**Table II:** Uropathogens in voided urine samples from study participants without symptoms or signs of urinary tract infection

Variable, n (%)	IUC in place on admission and history of catheterization past 4 weeks before admission, n=104	No IUC in place on admission and no history of catheterization past 4 weeks before admission, n=92	*P*-value
Significant bacteriuria for ASB^a^			0.018^d^
Yes	38 (37%)	19 (21%)	
No	66 (63%)	73 (79%)	
Significant bacteriuria according to recommendations for diagnosis of UTI^b^			0.015^d^
Yes	56 (54%)	33 (36%)	
No	48 (46%)	59 (64%)	
Bacteriuria, 10^3^–10^5^ CFU/mL^c^			0.847
Yes	17 (16%)	14 (15%)	
No	87 (84%)	78 (85%)	
*Escherichia coli,* ≥10^3^ CFU/mL in voided urine samples			0.869
Yes	27 (26%)	22 (24%)	
No	77 (74%)	70 (85%)	
*Enterococcus* species in voided urine samples^b^			<0.012^d^
Yes	12 (12%)	2 (2%)	
No	92 (88%)	90 (98%)	

IUC Indwelling urethral catheter.

ASB Asymptomatic bacteriuria.

a Any primary, secondary or tertiary uropathogen in the amount of ≥10^5^ CFU/mL.

b Primary uropathogen ≥10^3^ CFU/mL, secondary uropathogen as single species ≥10^3^ CFU/mL, secondary uropathogen ≥10^5^ CFU/mL when not as single species, tertiary uropathogen as single species ≥10^5^ CFU/mL.

c Any primary, secondary or tertiary uropathogen in the amount of 10^3^–10^5^ CFU/mL.

d A two-sided *P*-value of <0.05 was considered as a statistically significant difference.

In the multivariable regression analysis, a history of catheterization was significantly associated with ASB after adjustment for confounders (OR 2.79, 95% CI 1.31–5.91). In the final model, diabetes (OR 2.17, 95% CI 1.04–4.54) and ongoing antibiotic treatment for other reasons than UTI (OR 0.14, 95% CI 0.05–0.46) were associated with ASB (Table III). Hosmer-Lemeshow test indicated acceptable goodness-of-fit of the adjusted model.

**Table III:** Variables associated with significant bacteriuria for ASB^a^

Explanatory variable	Univariable OR (95% CI)	*P*-value	Multivariable OR (95% CI)	*P*-value
Sex		0.071^b^		0.056
Woman	1.89 (0.95–3.79)		2.15 (0.98–4.71)	
Man	Reference		Reference	
Age in age groups		0.556	N/A	
<80 years	Reference			
≥80 years	1.21 (0.64–2.27)			
Diabetes Mellitus		0.099^b^		0.039^c^
Yes	1.73 (0.90–3.33)		2.17 (1.04–4.54)	
No	Reference		Reference	
IUC present on admission or at any time during the past 4 weeks before admission		0.016^b^		0.008^c^
Yes	2.21 (1.16–4.21)		2.79 (1.31–5.91)	
No	Reference		Reference	
Antibiotic treatment		<0.001^b^		0.001^c^
No antibiotic treatment on admission or within two weeks before urine sample	Reference		Reference	
Antibiotic treatment on admission	0.20 (0.07–0.60)		0.14 (0.05–0.46)	
No antibiotic treatment on admission, but within 2 weeks before urine sample	2.51 (1.06–5.94)		1.51 (0.59–3.87)	
Post-void residual urine volume		0.443	N/A	
≤200 mL	Reference			
>200 mL	0.75 (0.36–1.57)			

ASB Asymptomatic bacteriuria.

N/A Not applicable.

IUC Indwelling urethral catheter.

UTI Urinary tract infection.

a Primary uropathogen ≥10^3^ CFU/mL, secondary uropathogen as single species ≥10^3^ CFU/mL, secondary uropathogen ≥10^5^ CFU/mL when not as single species, tertiary uropathogen as single species ≥10^5^ CFU/mL.

b Variables with a *P*-value ≤0.2 in the univariable analyses were included in the multivariable analyses of binary logistic regression.

c Variables with a *P*-value ˂0.05 in the adjusted multivariable analyses were considered as statistically significant associations.

Among 196 patients, 89 had bacteriuria according to recommendations for diagnosis of UTI (Table II).

Overall, *Escherichia coli* and *Enterococcus* species were the dominating bacteria detected in urine samples (49/196, 25%, and 14/196, 7.1%, respectively). *Staphylococcus saprophyticus* was not found in any sample.

Presence of *Escherichia coli* was equally distributed among the two patient groups with and without history of catheterization (27/104 vs 22/92, *P=*0.869) (Table II). However, *Enterococcus* species was significantly more commonly detected in patients with a history of catheterization compared to patients without (12/104 vs 2/92, *P*˂0.012).

### Urinary tract infection in patients during remaining inpatient care

Follow-up on the occurrence of UTI during the remaining inpatient care at the departments of geriatric rehabilitation was only possible at one of two study sites where 124 patients were included. In total, five female patients with ASB acquired UTI during the remaining inpatient period after the initial sampling for ASB. Of those, three patients had diabetes. Furthermore, four patients had an IUC on admission and one patient had no history of catheterization (*P=*0.082). Among those with an IUC, the treatment period was less than two weeks. Time from voided urine sampling to antibiotic treatment for UTI varied from 2 to 5 days.

Due to the small number of events (5 patients with UTI), no regression analyses were performed.

## Discussion

The main findings of this study were that ASB was significantly more common in patients with an IUC on admission or having had an IUC at any time during the past four weeks before admission, and that catheterization was associated with ASB when adjusted for confounding factors.

The odds ratio of ASB were 2.7 times higher in patients with a history of catheterization (*P=*0.008), and two times higher with diabetes (*P=*0.039). Ongoing antibiotic treatment for other reasons than UTI had a protective effect against ASB.

In our study, we found a prevalence of 36.5% ASB in patients with a history of catheterization. The prevalence was not significantly different among those who were sampled directly after IUC-removal, one-three days after or four or more days after. In comparison, Kass [27] found that women with >10⁵ CFU/mL urine in the first urine sample commonly also had the same elevated level of bacteriuria in a second urine sample taken within 1–12 months. Similarly, Rodhe *et al.* [32] found that an ASB prevalence of 19% in non-institutionalized elderly aged ≥80 years remained after 6 and 18 months. We did unfortunately not have an opportunity to take follow-up samples from the participating patients, but as shown by Kass and Rodhe *et al.* [27,32] ASB persists over time.

Simran *et al.* [33] conducted a study on persisting ASB 48 hours after IUC-removal. Out of 18 patients who were asymptomatic during the entire duration of catheterization until 48 hours after IUC-removal, four revealed significant bacteriuria (4/18, 22%). We were, however, only able to ask the patients about the presence of symptoms at the time of inclusion in the study. This could be an explanation for the higher prevalence of ASB (38/104, 36.5%) in our study. The higher prevalence of ASB, may also be due to the higher median age (81 years) of our study patients.

Our overall prevalence of ASB was higher than found by Rodhe *et al.* [32] which could be due to that our patients were institutionalised in contrast to their non-institutionalised residents.

We found that diabetes was significantly associated with ASB. Furthermore, ASB was significantly more common in diabetic women than in men (*P=*0.030) as well as more common in diabetic women (16/31) compared to non-diabetic women (27/98) (*P*=0.017). This is in line with the finding of Boyko *et al.* [34] where they found a significantly higher risk for ASB in postmenopausal diabetic women treated with insulin than in non-diabetic women.

Antibiotic treatment may postpone IUC-associated bacteriuria for up to 14 days [35]. We excluded patients with ongoing antibiotic treatment for UTI at admission as well as patients with new signs and symptoms diagnosed as UTI as we wanted to rule out factors that could dispute true ASB, i.e., significant bacteriuria without symptoms. We did however not exclude patients with an ongoing antibiotic treatment for other reasons than UTI such as pneumonia, skin-tissue-skeletal infections, or unspecified septicaemia. This may have masked symptoms of UTI and could be a reason for the finding that ongoing antibiotic treatment for other reasons than UTI had a significantly protective effect against ASB.

Regardless of history of catheterization, *Escherichia coli* was the most common uropathogen detected. *Staphylococcus saprophyticus* was not found in any urine sample. This is line with the findings of a Swedish cohort study conducted by Eriksson *et al.* [36].

*Enterococcus* species is more commonly detected in catheterized patients [16,37]. Consistent with this, we found that bacteriuria caused by enterococci (as single species ≥10^3^ CFU/mL or in a quantity of ≥10^5^ CFU/mL if detected together with other species in voided urine culture) was significantly more common in patients with a history of catheterization (12/104, 11.5%) compared to those without (2/92, 2.2%) (*P=*0.012).

Out of the 124 patients' records reviewed for UTI (124/196, 63%) only five UTI-cases (5/124, 4.0%) were identified. The few UTI-cases detected may be due to that ongoing antibiotic treatment for other reasons than UTI had a protective effect against ASB in our study patients. Lack of data concerning UTI from the other study site may also have underestimated the total number of UTI-cases found in our study, although we believe that the incidence of UTI did not differ between the study sites.

We used a cut-off point of 200 mL for elevated PVR as this is the clinical cut-off point that triggers follow-up of the patient's ability to empty the urinary bladder with further PVR measurements during inpatient care. Using this cut-off point we found an elevated PVR among 16–31% of the patients. We could not find any significant association between PVR and ASB (*P*=0.443) nor between PVR and UTI (*P=*0.346).

The figure for elevated PVR is in accordance with the results from Omli *et al.* [38] who found a PVR of ≥200 mL in 15% (22/150) of nursing home residents. In that study no association between PVR and UTI was found either.

In a study by Barabas & Mölstad [39], a PVR of >150 mL was found in 7% of nursing home residents and no association with bacteriuria was demonstrated. The higher prevalence of PVR in our study might be explained by inferior mobilisation of the patients as many were admitted to the geriatric wards after orthopaedic surgery or stroke.

### Strengths and limitations

Our study is one of few that have investigated presence of ASB in relation to the removal of the IUC in a clinical context. Our findings provide a starting point for further investigations regarding persistence of ASB after IUC-removal and the risk of developing UTI.

Limitations to our study are that no patients were included during weekends and holidays due to limited staffing and that inpatients not speaking and understanding Swedish were excluded. Another limitation is the lack of follow-up regarding ASB-persistence. Furthermore, UTI-cases were identified retrospectively by reading patients' records and this was only carried out in one of the two participating study sites, which may have underestimated the number of true UTI-cases among the included patients. The UTI-incidence may be higher in patients with long-term catheterization (more than 4 weeks), but this is beyond the scope of our study.

## Conclusions

Catheterization is significantly associated with ASB in geriatric inpatients, also after IUC-removal. *Enterococcus* species was more commonly found in patients with history of catheterization, which indicates that the colonisation of the urinary tract with enterococci caused by IUC may persist for at least four weeks after IUC-removal.

## Author contributions

AK, AT, EJA, and KA have taken part in conceptualisation, design of methodology, analysing of data, and writing of manuscript. AK has coordinated and administered the data collection. AK and EJA performed the statistical analyses. AT administered the project. AK, AT, EJA, and KA read and approved the final version.

## Funding statement

This research did not receive any specific grant from funding agencies in the public, commercial, or not-for-profit sectors.

## Conflict of interest statement

The authors have no conflict of interests to declare.
